# Negative impact of nurses’ fear of COVID-19: the moderating role of implementation of knowledge management

**DOI:** 10.3389/fpubh.2024.1426408

**Published:** 2024-07-25

**Authors:** Li-Chuan Chu

**Affiliations:** ^1^School of Health Policy and Management, Chung Shan Medical University, Taichung City, Taiwan; ^2^Department of Medical Education, Chung Shan Medical University Hospital, Taichung City, Taiwan

**Keywords:** nurses, fear, COVID-19, intention to leave the occupation, emotional labor, surface acting, deep acting, knowledge management

## Abstract

**Background:**

During the COVID-19 pandemic, nurses encountered substantial infection risks and psychological strain, which severely affected their emotional well-being, professional attitudes, and job performance. This study investigated the impact of nurses’ fear of COVID-19 on their intention to leave the occupation and emotional labor as well as the moderating role of the implementation of knowledge management on these primary variables.

**Methods:**

To mitigate common method bias, this research adopted a two-phase questionnaire approach, targeting nurses at a medical center in central Taiwan. In the first phase, 300 copies of questionnaire were distributed for participants to complete self-assessment surveys covering fear of COVID-19, knowledge management implementation, and demographic information. After 1 month, the participants were invited to complete a follow-up questionnaire, focusing on the intention to leave the occupation and emotional labor. The questionnaire was conducted from June to July 2022. Through this two-phase distribution method, after exclusion of invalid responses, a total of 288 valid responses were collected, resulting in a response rate of 96%. The proposed hypotheses were verified using hierarchical regression conducted with SPSS version 25.0.

**Results:**

The findings indicated that nurses’ fear of COVID-19 was significantly and positively associated with their intention to leave the occupation and surface acting, but negatively associated with their deep acting. Moreover, the implementation of knowledge management significantly moderated the positive relationship among fear of COVID-19, intention to leave the occupation, and surface acting. A robust knowledge management system weakened the positive association among fear of COVID-19, intention to leave the occupation, and surface acting.

**Conclusion:**

In summary, nurses’ fear of COVID-19 may increase their tendency to leave the nursing profession and engage in more surface acting and less deep acting. However, effective knowledge management practices can mitigate these adverse effects. Hospitals can thus establish and employ comprehensive knowledge management systems to enhance nurses’ resilience and help alleviate their fear of future pandemics and their potential negative repercussions.

## Introduction

The outbreak of the severe and highly contagious COVID-19 at the end of 2019 has profoundly affected the global community due to its high transmission and fatality rates. As of the end of March 2024, approximately 770 million people had been infected, with over 7 million succumbing to it (1). Nurses, serving as frontline caregivers in the battle against infectious diseases, are frequently exposed to patients, thus facing an elevated risk of infection (2). Early pandemic data revealed that from January 2020 to May 2021, an estimated 115,000 health-care workers worldwide died due to COVID-19 (3). This reality inevitably affects the psychological well-being and work performance of nurses (4). Throughout the pandemic, nurses have commonly experienced a fear of contracting COVID-19, worrying about both personal infection and the potential transmission of the virus to their loved ones (5). Such psychological fear triggers numerous negative effects, including depression (6), compromised mental health, increased work-related stress and burnout (7).

The fear stemming from the pandemic may drive nurses to consider leaving their nursing roles for positions with lower infection risks and comparatively simpler duties, potentially exacerbating the shortage of nursing personnel. Moreover, the fear surrounding the epidemic might impede their ability to effectively manage and convey emotions appropriately during interactions with patients or their families. This requisite for managing emotions in professional settings is referred to as emotional labor. In other words, the fear of COVID-19 among nurses may influence their display of emotional labor. Drawing from the perspectives of conservation of resources theory and affective event theory, this study investigated the impact of COVID-19 fear on nurses’ intention to leave the occupation and emotional labor during the pandemic.

Effectively mitigating nurses’ fear surrounding COVID-19 and its adverse impacts should be a paramount concern for hospitals. Taiwan, having experienced the repercussions of the Severe Acute Respiratory Syndrome (SARS) outbreak in 2003, took decisive actions to establish preventive measures against infectious diseases (8). In this context, knowledge management played a crucial role. By fostering the accumulation and dissemination of knowledge, governments can devise effective epidemic prevention policies, hospitals can implement prompt response measures, and the general public can acquire accurate concepts regarding epidemic prevention. Previous research has indicated that greater knowledge of COVID-19 is associated with less fear of COVID-19 (9). Therefore, when nurses effectively apply knowledge management to access information and skills related to the epidemic, it might weaken the adverse effects of the fear of COVID-19. This study examined the implementation of knowledge management as a moderating variable to investigate its impact on the relationship between nurses’ fear of COVID-19 and their intention to leave the occupation and emotional labor.

### Nurses’ fear of COVID-19 and their intentions to leave the occupation

The term “coronaphobia” has surfaced to encapsulate the fear surrounding COVID-19 and the associated physiological, psychological, and cognitive reactions it triggers, attributed to the virus’ high transmissibility and mortality rates (10). Nurses, in particular, face substantially higher risks of contracting COVID-19 compared with the general population, leading to anxieties about exposure during patient care and the inadvertent transmission of the virus to loved ones (4). Various factors such as patient deaths due to the virus, insufficient training in pandemic prevention, lack of managerial support, and shortages of medical equipment and protective gear have been identified to exacerbate these fears (2). Working within such environments, nursing professionals may increasingly contemplate leaving their profession for alternative career paths (11). Moreover, the number of new entrants willing to join the nursing profession may decline. In a qualitative study on nursing students in South Korea, Dos Santos (12) discovered that although many initially chose to study nursing for economic reasons and social status, the risks and changes brought about by the COVID-19 pandemic led many to plan to abandon the nursing field after graduation and pursue other careers.

Occupational turnover intentions refer to an individual’s inclination to transition from their current professional position to another field, and serve as a direct predictor of occupational turnover (13). The attrition of nurses not only represents a loss in the investment made in nursing education (14) but also adversely affects the quality of care and patient safety due to the staffing shortages (15). The shortage of nurses is a widespread concern globally (14). Recognizing the factors influencing nurses’ intentions to leave the occupation and implementing effective strategies accordingly can help mitigate attrition rates. Studies have indicated several reasons for nurses opting to leave the profession, including excessive workloads, strained interpersonal relationships with colleagues, limited avenues for advancement and professional growth, and deficiencies in leadership and communication (16). Additionally, the lethal risk posed by global pandemics contributes to this phenomenon (11).

Fear represents an emotional response evoked in individuals when confronted with a perceived threat, which then triggers various behavioral responses aimed at mitigating risks, including avoidance or coping strategies (17). According to the affective events theory (18), emotions of employees are influenced by work-related incidents, which in turn affect their work attitudes and behavioral performance. Therefore, when nurses perceive the threat posed by COVID-19 and experience fear, they may contemplate leaving their nursing jobs to evade potential risks. From the perspective of conservation of resource theory (19, 20), individuals strive to safeguard and conserve valuable resources, experiencing stress and distress in the face of resource loss or threat. During the pandemic, nurses’ fear of COVID-19 serves as a source of stress (21). In the absence of positive resources from the hospital, such as adequate personal protective equipment or emotional support and assistance from supervisors and colleagues (4), a continuous depletion of their personal resources can occur (22), leading to various negative psychological symptoms, such as compromised mental health (7). To avoid resource depletion, nurses experiencing heightened fear of COVID-19 may be more inclined to consider leaving the profession. Past research (11) has confirmed that the stronger the nurses’ fear of COVID-19 is, the greater their tendency to leave the nursing profession is.

Based on the above discussion, this study posited the following hypothesis:

*Hypothesis 1*: The greater the nurses’ fear of COVID-19 is, the higher their intention to leave the occupation becomes.

### Nurses’ fear of COVID-19 and its impact on emotional labor

In addition to potentially increasing nurses’ tendency to leave the profession, the fear of COVID-19 may affect their performance of emotional labor. Nurses are often expected to exhibit qualities of compassion and care, necessitating them to uphold a composed emotional and attitudinal disposition toward patients, attentively listen to patients’ concerns, and demonstrate genuine care for their well-being irrespective of their own emotional states (23). However, the threat posed by COVID-19 may induce fear and anxiety among nurses, posing challenges in maintaining stable emotional states and professional conduct, consequently impeding their execution of emotional labor.

Hochschild introduced the concept of emotional labor in 1983, highlighting the work behavior of flight attendants who are required to constantly smile to make customers feel safe and to ensure a positive service experience. Emotional labor can be defined as the management of internal emotions and external emotional expressions during interpersonal interactions in the workplace, enabling individuals to conform to the emotional display rules set by the organization (24). Emotional labor is categorized into surface acting and deep acting. Surface acting refers to employees, despite experience a disconnect between their internal emotions and the outward emotional expression required by their job, adjusting their facial expressions or tone of voice to adhere to the organization’s emotional norms, while concealing or suppressing their genuine internal feelings (24). This incongruence between internal emotions and external expressions leads to emotional dissonance. Conversely, deep acting involves employees aligning their internal emotions with their external expressions through cognitive adjustments, thereby minimizing the occurrence of emotional dissonance (24).

Research has established that nurses’ surface acting has a negative association with well-being (25) and nurse–patient relationships (26). By contrast, nurses’ deep acting is positively associated with job satisfaction, job performance (27), and nurse–patient relationships (26) and negatively associated with emotional exhaustion (28).

Numerous factors can influence emotional labor, including employees’ compassion fatigue (29), professional identity (28). However, the impact of the fear of COVID-19 on nurses’ emotional labor performance remains uncertain. According to the conservation of resources theory proposed by Hobfoll (19), individuals are motivated to conserve, safeguard, and enhance the resources they value. When confronted with resource scarcity, individuals employ strategies to conserve their resources for self-protection (20). Despite potentially experiencing the fear of COVID-19, nurses must exhibit appropriate emotions when interacting with patients, aligning with hospital norms and external expectations. However, due to a shortage of emotional resources and inadequate external support, they may tend to engage more in surface acting and less in deep acting, aiming to mitigate excessive depletion of emotional resources. This is because, under conditions of resource scarcity, suppressing emotions through surface acting is comparatively simpler than adjusting internal feelings through deep acting (28). Studies have confirmed that nurses with high levels of fear of COVID-19 are more susceptible to emotional exhaustion (22). Furthermore, Liu et al. (28) indicated that nurses experiencing emotional exhaustion tend to resort to surface acting. Hur and Shin (30) also confirmed that employees experiencing resource depletion are more inclined to engage in surface acting while simultaneously reducing deep acting.

According to the affective event theory (18), workplace events trigger emotions in employees, which in turn influence their behavioral performance. The outbreak of COVID-19 has instilled fear among nurses, making it difficult for them to align their cognitive processes with the display of empathy and care during emotional labor. This challenge arises because individuals’ negative emotions contradict the emotional expression guidelines required by the organization, thereby increasing the difficulty in adjusting internal feelings (31). Lam and Chen (32) demonstrated that individuals experiencing negative emotions tend to resort to surface acting while reducing deep acting. Du et al. (33) discovered that employees tend to engage in more surface acting when experiencing anxiety induced by the pandemic.

Based on the above discussion, this study posited the following hypotheses:

*Hypothesis 2-1*: The greater the nurses fear of COVID-19 is, the more they engage in surface acting.

*Hypothesis 2-2*: The greater the nurses’ fear of COVID-19 is, the less they engage in deep acting.

### Relationship between nurses’ fear of COVID-19 and their intention to leave the occupations and emotional labor: the moderating role of the implementation of knowledge management

During the pandemic, the potential adverse effects of nurses’ fear of COVID-19, such as increased intentions to leave their occupation and a propensity toward surface acting over deep acting, have been substantial. This necessitates exploring effective measures that can be implemented by hospital authorities to mitigate these negative effects.

Following the SARS outbreak in 2003, Taiwan established a public health response system. In 2004, the National Health Command Center was founded, which was tasked with coordinating responses to epidemic diseases and emergency medical situations (8). Starting from 2005, hospitals at regional levels and above were mandated to undergo annual infection control assessments. Additionally, the National Health Command Center planned 134 isolation hospitals and 1,100 negative pressure isolation rooms to address future infectious disease outbreaks in a timely manner (34). With the onset of the COVID-19 outbreak, Taiwan instituted the Central Epidemic Command Center, implementing measures such as border controls, isolation protocols for suspected cases, allocation of medical resources, and dissemination of public information (8). By using the expertise accumulated since 2003 and through knowledge accumulation and sharing, the government facilitated hospital knowledge management. This aided in a rapid response to the challenges posed by the COVID-19 pandemic, instilling confidence in the public regarding hospital visits.

Knowledge management involves the coordination of individuals, processes, and technology, enabling organizations to successfully create, acquire, share, and apply knowledge (35). Knowledge is typically categorized into explicit knowledge and tacit knowledge. Explicit knowledge refers to information that is systematically recorded through methods such as sound, images, and text, thus enabling easy storage and shareability. Tacit knowledge refers to subjective knowledge that is difficult to articulate through words or images and is closely linked to individual behaviors and contexts, including mental models, beliefs, and values (35). The processes involved in knowledge management comprise knowledge discovery, knowledge capture, knowledge sharing, and knowledge application (36). Through the implementation of knowledge management, organizations can establish and organize relevant knowledge, enabling members to effectively access, utilize, store, share, and innovate. This aids in achieving objectives such as experience transfer, reduction of operational costs, enhancement of response capacities, and preservation of competitive advantage (35). Knowledge management enables hospitals to optimize resource utilization and improve the quality of patient care (36).

Nurses function as knowledge workers who use medical knowledge and professional expertise to navigate complex challenges and make informed decisions (37). Particularly when addressing infectious diseases, nurses must acquire a substantial amount of relevant knowledge, often through avenues such as journal research or learning from experienced colleagues (38). For example, Taiwan’s response to the SARS epidemic in 2003 facilitated health-care professionals in swiftly addressing the COVID-19 outbreak, effectively implementing preventive measures (39). The understanding of pandemic-related knowledge influences nurses’ perceptions of risk and informs prevention strategies (38). Therefore, the hospital’s knowledge management system plays a critical role in this regard.

Nurses often experience fear primarily due to their limited experience and professional knowledge in handling crisis situations (2). However, if nurses have access to a well-implemented knowledge management system within the hospital, coupled with a culture that encourages knowledge sharing, they can improve their understanding of pandemic-related knowledge and receive timely information, thus alleviating their fears (2). Studies have reported that enhancing nurses’ knowledge about diseases such as HIV/AIDS can effectively reduce their fear of these illnesses and increase their willingness to care for patients affected by them (40). From the perspective of the conservation of resources theory, individuals must invest resources to prevent the loss of existing resources, including enhancing skills and knowledge to cope with competitive work environments (20). The effective implementation of knowledge management can enhance nurses’ ability to respond to COVID-19, increasing their personal resources, mitigating the fear of COVID-19, and consequently mitigating potential negative impacts. Mubarak et al. (9) reported that the higher the nurses’ level of knowledge about COVID-19 is, the lower their fear of the virus becomes. This underscores the importance of enhancing nurses’ understanding of pandemic information, such as transmission routes, preventive measures, the use of protective equipment, infection protocols, and potential treatment options, in reducing their anxiety and enhancing their confidence in fulfilling their responsibilities.

In summary, nurses with a higher level of implementation of knowledge management experience lower fear of COVID-19 and fewer potential negative impacts. In other words, the level of implementation of knowledge management effectively moderates the relationship between nurses’ fear of COVID-19 and both their intention to leave the occupation s and emotional labor.

Based on the literature review, this study proposed the following hypothesis:

*Hypothesis 3-1*: The implementation of knowledge management effectively moderates the positive relationship between nurses’ fear of COVID-19 and their intention to leave the occupation.

*Hypothesis 3-2a*: The implementation of knowledge management effectively moderates the positive relationship between nurses’ fear of COVID-19 and surface acting.

*Hypothesis 3-2b*: The implementation of knowledge management effectively moderates the negative relationship between nurses’ fear of COVID-19 and deep acting.

## Materials and methods

### Study design

To mitigate the risk of common method bias (41), a two-stage questionnaire approach was adopted in this study. In the first stage, participants’ levels of fear regarding COVID-19, the implementation of knowledge management, and demographic information were assessed. One month later, the same cohort of participants was invited to complete the second stage of the self-assessment questionnaire, which included items on intention to leave the occupation and emotional labor. Prior to the distribution of the questionnaire copies, approval was obtained from the institutional review board, and consent was obtained from the research site. Copies of the questionnaire were then distributed to the head nurses of various departments in the hospital, who in turn distributed them to the nurses in their respective units. To minimize response bias, the questionnaire was completed anonymously. Because of the two-stage design of the study, the head nurses were instructed to mark the copies of questionnaire and envelopes for the second stage with the same identification number. Upon completing their questionnaire responses at different stages, the participants were instructed to place their completed responses in sealed envelopes and return them to their head nurse. Finally, the head nurses collected all the returned envelopes and placed them in a larger envelope, which was then collected by the research team from the hospital.

### Participants

The study targeted nurses across emergency departments, intensive care units, infectious disease departments, negative pressure isolation wards or specialized wards, general wards, and outpatient departments of a medical center in central Taiwan. The inclusion criterion was working as a nurse during the COVID-19 pandemic. The exclusion criterion was refusal to participate. Data collection was conducted from June to July, 2022. Through convenience sampling, 300 copies of questionnaire were distributed in the first stage. One month later, the same group of personnel responded to the second-stage questionnaire. After excluding invalid questionnaire responses due to unsuccessful matching of the two-stage questionnaire and incomplete data, a total of 288 valid responses were obtained, resulting in a response rate of 96%. Regarding demographic characteristics, the majority of participants were female, accounting for 94.4% (272 individuals). In terms of marital status, single participants were the most predominant, comprising 67.4% (194 individuals). Regarding education level, participants with a university degree constituted the largest proportion, totaling 248 or 86.1%. Regarding positions, nonmanagerial staff comprised the majority at 96.2% (277 individuals). In terms of departmental affiliation, the general ward had the highest number of participants, with 40.3% (116 individuals). Regarding contact with or care for confirmed individuals with COVID-19, those who occasionally had contact constituted the largest group, with 56.7% (170 individuals). Additionally, the average age of the participants was 32.54 years, with an average tenure at the current hospital of 8.61 years.

### Ethical considerations

In adherence to ethical standards, the protocol for this research was approved by the Institutional Review Board at Chung Shan Medical University Hospital, Taiwan (IRB No.: CS1-22061). Prior to participating, all the participants provided written informed consent. They were briefed on the study’s objectives and assured of their right to withdraw from the study at any point. Moreover, measures were implemented to ensure the confidentiality of their survey data.

### Measures

Fear of COVID-19 was assessed using the Fear of COVID-19 Scale developed by Ahorsu et al. (42), comprising seven items. An example item was “I am afraid of losing my life because of coronavirus-19.” Respondents rated their agreement with each item on a six-point Likert scale, ranging from 1 (strongly disagree) to 6 (strongly agree), with higher scores indicating heightened fear of COVID-19. Previous research reported favorable reliability (*α* = 0.82) and validity of the scale (42).

The intention to leave the occupation was assessed using the scale developed by Meyer et al. (43), comprising three items. An example item was “I frequently thought about leaving the nursing profession.” Respondents indicated their agreement with each item on a six-point Likert scale, ranging from 1 (strongly disagree) to 6 (strongly agree), with higher scores suggesting a greater tendency to leave the nursing profession. Previous research reported favorable reliability (*α* = 0.83) and validity of the scale (43).

Emotional labor was assessed using the emotional labor scale by Diefendorff et al. (44), which comprises two dimensions: surface acting and deep acting. The surface acting dimension had seven items. An example item was “I put on a ‘mask’ in order to display the emotions I need for the job.” The deep acting dimension had four items. An example item was “I try to actually experience the emotions that I must show to patients.” Participants rated their agreement with each item on a six-point Likert scale, ranging from 1 (strongly disagree) to 6 (strongly agree), with higher scores on each dimension indicating a higher degree of emotional labor. Previous research reported favorable reliability (*α* = 0.92 for surface acting and 0.85 for deep acting) and validity of the scale (44).

The implementation of knowledge management was assessed using the scale developed by Choi (45) and adjusted by Lee (46), which comprises 23 items. An example item was “I will share ideas and resources with members.” Participants rated their agreement with each item on a six-point Likert scale, ranging from 1 (strongly disagree) to 6 (strongly agree), with higher scores indicating a greater level of application of knowledge management by employees. Previous research reported favorable reliability (*α* = 0.93) and validity of the scale (46).

In accordance with previous findings, which suggest that an employee’s organizational tenure may influence both intention to leave the occupation (13) and emotional labor (47), this study included organizational tenure as a control variable.

### Data analysis

The questionnaire data collected in this study were analyzed using SPSS version 25.0. Descriptive statistics were employed to assess the demographic characteristics of the nurses. A correlation analysis was employed to explore the relationships among fear of COVID-19, intention to leave the occupation, emotional labor, and the implementation of knowledge management. Hierarchical regression analysis was conducted to test the hypotheses of this study. The level of statistical significance was set at *p* < 0.05.

## Results

Following the survey administration, the reliability of the scales was assessed. The α coefficient for fear of COVID-19, implementation of knowledge management, intention to leave the occupation, surface acting, and deep acting was 0.92, 0.97, 0.93, 0.92, and 0.93, respectively. These results indicate that the scales used in this study demonstrate high reliability.

Table 1 presents the results of the correlation analysis, revealing significant associations among fear of COVID-19, implementation of knowledge management, intention to leave the occupation, surface acting, and deep acting. Specifically, fear of COVID-19 exhibited a significant positive correlation with intention to leave the occupation and surface acting (*r* = 0.43, *p* < 0.01; *r* = 0.33, *p* < 0.01, respectively). Additionally, fear of COVID-19 demonstrated a significant negative correlation with deep acting and implementation of knowledge management (*r* = −0.24, *p* < 0.01; *r* = −0.12, *p* < 0.05, respectively). Moreover, implementation of knowledge management exhibited a significant negative correlation with intention to leave the occupation (*r* = −0.33, *p* < 0.01) and positive correlation with deep acting (*r* = 0.21, *p* < 0.01).

**Table 1:** Descriptive statistics and intercorrelations among study variables.

Variable	1	2	3	4	5
1. Fear of COVID-19	(0.92)				
2. Implementation of knowledge management	−0.12*	(0.97)			
3. Intention to leave the occupation	0.43**	−0.33**	(0.93)		
4. Surface acting	0.33**	−0.06	0.38**	(0.92)	
5. Deep acting	−0.24**	0.21**	−0.16**	0.08	(0.93)
Mean	2.92	4.42	3.47	3.82	4.33
SD	1.09	0.75	1.38	0.98	0.81

Cronbach’s alphas appear on the diagonal. *p < 0.05; **p < 0.01.

To avoid potential issues related to multicollinearity, this study calculated the interaction term using standardized z-scores. Fear of COVID-19 and implementation of knowledge management were standardized before conducting regression analysis. Table 2 presents the results of the hierarchical regression analysis. With organizational tenure considered as a control variable, it significantly and negatively predicted intention to leave the occupation (*β* = −0.14, *p* < 0.05). Upon inclusion of fear of COVID-19 in this model (step 2). The results revealed that fear of COVID-19 significantly and positively predicted intention to leave the occupation (*β* = 0.44, *p* < 0.01), indicating that the greater the nurses’ fear of COVID-19 is, the higher their intention to leave the occupation is. Thus, Hypothesis 1 was supported. Furthermore, fear of COVID-19 significantly and positively predicted surface acting (*β* = 0.33, *p* < 0.01) and significantly and negatively predicted deep acting (*β* = −0.25, *p* < 0.01), indicating that the greater the nurses’ fear of COVID-19 is, the higher their engagement in surface acting is and lower their engagement in deep acting is. These findings support Hypothesis 2-1 and Hypothesis 2-2.

**Table 2:** Moderating effect of knowledge management implementation on the relationship between fear of COVID-19, intention to leave the occupation, and emotional labor.

Dependent variables	Intention to leave the occupation				Surface acting				Deep acting			
Independent variables	Step 1	Step 2	Step 3	Step 4	Step 1	Step 2	Step 3	Step 4	Step 1	Step 2	Step 3	Step 4
	Beta	Beta	Beta	Beta	Beta	Beta	Beta	Beta	Beta	Beta	Beta	Beta
Organizational tenure	−0.14*	−0.18**	−0.16**	−0.17**	0.05	0.02	0.02	0.01	0.02	0.04	0.03	0.03
Z fear of COVID-19		0.44**	0.41**	0.40**		0.33**	0.32**	0.32**		−0.25**	−0.22**	−0.22**
Z implementation of knowledge management			−0.27**	−0.26**			−0.02	−0.02			0.18**	0.18**
Z fear of COVID-19 × Z implementation of knowledge management				−0.17**				−0.11*				0.04
*R* ^2^	0.02	0.21	0.28	0.31	0.00	0.11	0.11	0.12	0.00	0.06	0.09	0.09
Adjusted *R*^2^	0.02	0.21	0.28	0.30	−0.00	0.10	0.10	0.11	−0.00	0.05	0.08	0.08
Δ*R*^2^	0.02*	0.19**	0.07**	0.03**	0.00	0.11**	0.00	0.01*	0.00	0.06**	0.03**	0.00
*F*	5.96	38.47	37.47	31.83	0.57	17.01	11.36	9.65	0.12	9.13	9.59	7.29

*N* = 288, *p < 0.05, **p < 0.01.

**Figure 1:**
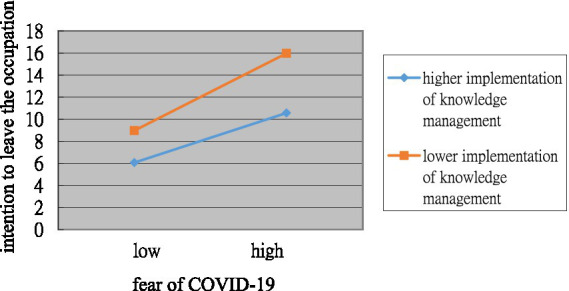
Plot of the interaction between fear of COVID-19 and implementation of knowledge management on intention to leave the occupation.

**Figure 2:**
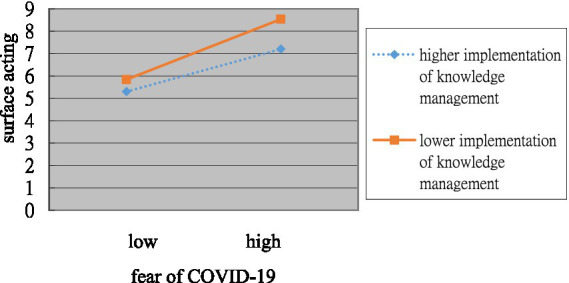
Plot of the interaction between fear of COVID-19 and implementation of knowledge management on surface acting.

When the implementation of knowledge management was introduced into the model (step 3), it significantly and negatively predicted intention to leave the occupation (*β* = −0.27, *p* < 0.01) and significantly and positively predicted deep acting (*β* = 0.18, *p* < 0.01). Furthermore, upon adding the interaction between fear of COVID-19 and the implementation of knowledge management into this model (step 4), it negatively predicted intention to leave the occupation (*β* = −0.17, *p* < 0.01) and surface acting (*β* = −0.11, *p* < 0.05) but did not significantly predict deep acting (*β* = 0.04, *p* = 0.50), therefore Hypothesis 3.3 was not supported. To facilitate comprehension, Figures 1, 2 present the moderating effects. Initially, the sample was divided into two groups based on high and low levels of implementation of knowledge management, delineated by one standard deviation above and below the mean. The impact of fear of COVID-19 on intention to leave the occupation was weaker (*β* = 0.35, *p* < 0.05) for employees who reported a higher knowledge management implementation than for those who reported a lower knowledge management implementation (*β* = 0.69, *p* < 0.01). Thus, Hypothesis 3-1 was supported.

In the group with a low level of knowledge management implementation, fear of COVID-19 exhibited a significant positive association with surface acting (*β* = 0.41, *p* < 0.05). However, in the high implementation group, the impact of fear of COVID-19 on surface acting was positive (*β* = 0.20), but not significant (*p* > 0.05). This suggests that a high level of knowledge management implementation tends to weaken the positive relationship between fear of COVID-19 and surface acting, although this moderating effect did not reach statistical significance. Conversely, in the low implementation group, the positive relationship between fear of COVID-19 and surface acting was further strengthened. Thus, Hypothesis 3-2a was partially supported.

## Discussion

This study investigated the impact of nurses’ fear of COVID-19 on their intention to leave the occupation and emotional labor during the pandemic period as well as the moderating effect of the implementation of knowledge management. The results revealed that nurses’ fear of COVID-19 significantly and positively predicts their intention to leave the occupation. This result is consistent with the findings of Mehra et al. (11), aligning with the conservation of resources theory. Because the ongoing threat of the pandemic depletes personal resources (22), nurses who are more fearful of COVID-19 are more likely to consider leaving their nursing jobs. Nurses who experience heightened fear of COVID-19 may be more inclined to contemplate leaving their profession due to concerns about the associated risks, such as infection transmission. Furthermore, the study discovered that fear of COVID-19 significantly and positively predicts surface acting and negatively predicts deep acting among nurses. These results are in line with the conservation of resources theory, suggesting that individuals tend to conserve resources when faced with resource scarcity (20). Fear of COVID-19 leads to the depletion of internal resources (22), leading to increased surface acting and decreased deep acting (30). These findings are also consistent with the affective event theory (18). Fear of COVID-19 can be considered a negative emotion that causes nurses’ internal feelings to diverge from the emotional expression rules required by the organization, making it difficult to adjust their internal feelings, thus resulting in more surface acting and less deep acting (32).

Moreover, the study underscored the moderating influence of knowledge management implementation on the relationship between nurses’ fear of COVID-19 and both their intention to leave the occupation and surface acting tendencies. This finding corroborates previous research by Mubarak et al. (9), suggesting that nurses who possess a deeper understanding of COVID-19 are better equipped to alleviate their fear, anxiety, and stress. In other words, a higher level of knowledge management implementation among nurses increases their likelihood of accessing pandemic prevention and treatment care information through the knowledge management system of the hospital or through knowledge exchange with colleagues. This reduces fear of COVID-19, mitigates its adverse impact, lowers their intention to leave the occupation, and reduces surface acting.

Regarding demographic variables, this study discovered that organizational tenure negatively predicts nurses’ intention to leave the occupation, consistent with the findings of Van der Heijden et al. (13). Longer organizational tenure indicates that nurses who have devoted more time to their profession are more familiar with and proficient in their current job roles and have less time and energy to learn new skills in a different field. Thus, they are less likely to consider changing professions. Furthermore, tenure did not significantly predict the emotional labor of nurses. Studies have reported that employees with longer tenure engaged in more deep acting and less surface acting (47). This may be because nurses gradually develop a professional identity when they are accumulating experience (48). This identity helps them to express organizationally sanctioned emotions without having to fake them, leading to more deep acting and less surface acting (28). However, the fear induced by the pandemic could have overshadowed the effect of tenure on emotional labor, resulting in increased surface acting and reduced deep acting among nurses regardless of their tenure.

### Limitations and future directions

Despite striving for methodological rigor, this study encountered several limitations. First, the primary variables were measured through self-assessment, which, despite being anonymous, may be susceptible to response bias due to social desirability. Nurses might be reluctant to express their genuine thoughts about COVID-19, potentially affecting their responses. Additionally, data collection was conducted from May to July 2022, and nurses’ fear levels may have fluctuated as the pandemic evolved. In addition, nursing staff who experienced extreme fear of the epidemic could have left nursing early in the pandemic. Therefore, future studies could adopt a longitudinal research design to investigate potential changes in nurses’ fear responses at different stages of the pandemic’s progression. Moreover, this study focused exclusively on nurses from a medical center in central Taiwan, limiting the generalizability of the findings to nurses in other regions or countries. Future studies could broaden the scope by including medical centers from various regions within Taiwan or even from different countries to enhance the external validity of the results. Alternatively, researchers could explore variations among different types of health-care institutions, such as medical centers, regional hospitals, district hospitals, or clinics, to assess potential differences in nurses’ fear responses, intention to leave the occupation, emotional labor, or the implementation of knowledge management during the pandemic.

This study confirmed that fear of COVID-19 exacerbates nurses’ intention to leave the profession, increases surface acting, and reduces deep acting. Studies have also indicated that fear of the pandemic leads to increased work-related stress and burnout among nurses (7). This highlights the necessity of understanding the causes of pandemic-related fear, such as a hospital’s preparedness with protective equipment and individual personality traits (2). By understanding the roots of pandemic fear, hospital administrators can develop relevant strategies to prevent or mitigate the fear experienced by nurses.

### Recommendations for practice

Nurses’ intention to leave the occupation is closely related to the problem of nursing shortages (15), whereas emotional labor is associated with their work attitudes and performance (26). Both are critical concerns for hospitals. The study findings indicated that fear of COVID-19 increases nurses’ intention to leave the occupation and surface acting, but reduces deep acting. To address this, health-care institutions are advised to prioritize nurses’ emotional well-being during the pandemic to alleviate their fear and discomfort, ensuring that they can maintain their professional caregiving capabilities.

Hospitals can enhance their understanding of the factors contributing to nurses’ fear and implement tailored interventions accordingly. One primary concern for nurses may revolve around the perceived risk of infection and inadequate protective gear availability (2). Therefore, hospitals should ensure a consistent supply of essential personal protective equipment, such as masks and protective clothing, regularly monitoring inventory levels and restocking as required (7). Additionally, implementing electronic devices to replace manual tasks and reducing unnecessary contact opportunities can further mitigate infection risks. Moreover, nurses may feel anxious about their preparedness to handle complex situations during the pandemic, such as unfamiliarity with protective equipment and related care protocols (2). Hospitals should respond by establishing robust care protocols and offering targeted technical training to equip nurses with the necessary skills and confidence to navigate challenges arising during the pandemic (4). Most importantly, hospitals must prioritize creating a supportive environment where nursing supervisors actively engage with nurses, addressing their work-related concerns and offering avenues for psychological support. Providing access to counseling services and dedicated spaces for rest and relaxation are essential support measures that foster a positive and supportive work environment (6).

The study findings underscored the pivotal role of knowledge management in alleviating nurses’ fear of COVID-19 and mitigating its adverse effects. Therefore, hospitals must strengthen their knowledge management systems across various departments. By standardizing operational procedures and implementing an integrated knowledge management system, a comprehensive database for educational training materials can be created on cloud systems, containing information on pandemic precautions for staff, ward traffic management protocols, surveillance and warning systems, and guidelines for donning and doffing isolation gear. Additionally, documenting training sessions and encouraging knowledge sharing among experienced nurses can facilitate knowledge transfer. For instance, in response to a large number of COVID-19 cases, many frontline nurses have developed innovative care approaches amid equipment and medication shortages (49). Such valuable insights can be curated and shared through the knowledge management platform, benefitting medical facilities nationwide. Leveraging online platforms for knowledge exchange not only promotes social distancing to mitigate infection risks but also enhances learning resource accessibility and participation rates among nurses (50).

Supervisors play a pivotal role in the knowledge management system (35). Their responsibilities encompass facilitating the acquisition of professional knowledge, providing supervision, offering support, and fostering a culture of knowledge sharing (46). Therefore, hospitals must prioritize the development of leadership competencies among supervisors, enabling them to serve as role models, inspire subordinates to broaden their knowledge base, enhance their expertise, and consistently provide constructive professional feedback.

By refining and effectively implementing the knowledge management system, hospitals can ensure that nurses consistently have access to up-to-date and comprehensive information regarding the pandemic. This includes insights into the virus’ characteristics, strategies for preventing transmission, patient management protocols, and the optimal utilization of hospital resources. Such accessibility to crucial knowledge can help alleviate the nurses’ fear of the virus, allowing them to focus on their caregiving responsibilities.

## Conclusion

Amid the surge of an epidemic, nurses play a crucial role. Their feelings of fear toward COVID-19 can influence the display of their emotional labor and potentially escalate their inclination to leave nursing roles. Hospitals should prioritize the establishment of a robust knowledge management system, promptly furnishing essential pandemic updates, aiding nurses in understanding pertinent information, thereby reducing their fears. Only a workplace with a sense of security can enable nurses to deliver high-quality care.

## Data availability statement

The raw data supporting the conclusions of this article will be made available by the authors, without undue reservation.

## Ethics statement

In adherence to ethical standards, the protocol for this research was approved by the Institutional Review Board at Chung Shan Medical University Hospital, Taiwan (IRB No.: CS1-22061). Prior to participating, all the participants provided written informed consent. They were briefed on the study’s objectives and assured of their right to withdraw from the study at any point. Moreover, measures were implemented to ensure the confidentiality of their survey data.

## Author contributions

L-CC: Conceptualization, Data curation, Formal analysis, Funding acquisition, Investigation, Methodology, Project administration, Resources, Software, Supervision, Validation, Visualization, Writing – original draft, Writing – review & editing.
